# Parallel reduction in expression, but no loss of functional constraint, in two opsin paralogs within cave populations of *Gammarus minus* (Crustacea: Amphipoda)

**DOI:** 10.1186/1471-2148-13-89

**Published:** 2013-04-23

**Authors:** David B Carlini, Suma Satish, Daniel W Fong

**Affiliations:** 1 Department of Biology, American University, Washington, DC, USA

## Abstract

**Background:**

*Gammarus minus*, a freshwater amphipod living in the cave and surface streams in the eastern USA, is a premier candidate for studying the evolution of troglomorphic traits such as pigmentation loss, elongated appendages, and reduced eyes. In *G. minus*, multiple pairs of genetically related, physically proximate cave and surface populations exist which exhibit a high degree of intraspecific morphological divergence. The morphology, ecology, and genetic structure of these sister populations are well characterized, yet the genetic basis of their morphological divergence remains unknown.

**Results:**

We used degenerate PCR primers designed to amplify opsin genes within the subphylum Crustacea and discovered two distinct opsin paralogs (average inter-paralog protein divergence ≈ 20%) in the genome of three independently derived pairs of *G. minus* cave and surface populations. Both opsin paralogs were found to be related to other crustacean middle wavelength sensitive opsins. Low levels of nucleotide sequence variation (< 1% within populations) were detected in both opsin genes, regardless of habitat, and dN/dS ratios did not indicate a relaxation of functional constraint in the cave populations with reduced or absent eyes. Maximum likelihood analyses using codon-based models also did not detect a relaxation of functional constraint in the cave lineages. We quantified expression level of both opsin genes and found that the expression of both paralogs was significantly reduced in all three cave populations relative to their sister surface populations.

**Conclusions:**

The concordantly lowered expression level of both opsin genes in cave populations of *G. minus* compared to sister surface populations, combined with evidence for persistent purifying selection in the cave populations, is consistent with an unspecified pleiotropic function of opsin proteins. Our results indicate that phototransduction proteins such as opsins may have retained their function in cave-adapted organisms because they may play a pleiotropic role in other important processes that are unrelated to vision.

## Background

A large number of higher taxa contain species restricted to subterranean habitats, and most of these species show convergent differentiation in a suite of features related to the absence of light [1,2]. These features, termed troglomorphy by Christiansen 1962 [3], include partial to complete loss of eyes and body pigment, elaborated extra-optic sensory structures, elongated appendages, and more slender body forms compared to related surface-dwelling taxa. The elaborated and elongated features are generally assumed to be adaptations to the aphotic environment from selection for enhanced extra-optic sensory performance and efficiency of movement, and are considered as textbook examples of evolution by means of natural selection. The reduction to loss of eyes and body pigment in subterranean species is part of a universal phenomenon of vestigialization to loss of structures common to the evolutionary history of most if not all phyletic lineages. Thus, the evolution of elaborated, and of even reduced features, is not exclusive to subterranean taxa, *e.g.*, the loss of hearing in moths on islands without bats [4]. The study of the evolution of troglomorphy in subterranean species is interesting because both elaborated and reduced features are exhibited by the same organism, have evolved independently and repeatedly across many taxa, and are clearly related to the consequences of a single overriding environmental constraint: total darkness.

The mechanism behind the evolutionary loss of eyes and body pigment is difficult to elucidate, not because it is difficult to explain, but because many of the proposed explanations are difficult to frame into testable hypotheses [1,4,5]. Hypotheses regarding the mechanism of such reductive evolution in subterranean species fall into three groups: non-adaptive, directly adaptive, and indirectly adaptive. The non-adaptive hypothesis invokes neutral mutation and drift and attributes no role to selection. Simply, features such as eyes and body pigment serve no function in darkness, thus mutations of structural loci underlying these traits are neutral and would accumulate over time, resulting in the breakdown of the feature. The direct adaptation hypothesis suggests that selection directly favors individuals with smaller eyes or reduced pigment *per se* in darkness, although it is difficult to envision the immediate selective advantage of smaller eyes or reduced pigment. Usually, such an advantage is couched in terms of energy savings by minimizing the cost of developing or maintaining a functionless structure. Fong 1989 [6], however, argued that this is simply part of the indirectly adaptive hypothesis because the selective advantage of the energy saved must come from spending the energy on enhancing the performance of other features in darkness. The non-adaptive and directly adaptive hypotheses assume independence of the sets of elaborated and reduced features. The indirectly adaptive hypothesis assumes a negative genetic correlation between the elaborated and reduced features through antagonistic pleiotropy, *i.e.*, that evolutionary reduction in features is a correlated response to selection for elaborated features.

Recent studies have shed light on the relative roles of selection compared to neutral mutation and drift in the reduction to loss of features in cave organisms. Jones et al. 1992 [7] used differences in morphology of paired, reproducing individuals with sexually matured but unpaired individuals to estimate the selection gradient of body size, eye size and antenna size in both surface and cave populations in the amphipod crustacean *Gammarus minus*. They showed that the intensity and direction of selection were similar among cave populations and similar among surface populations, but that selection favored smaller eyes in cave populations and larger eyes in spring populations. Cave species of the freshwater crab genus *Sundathelthusa* exhibit equally rapid rates of reduction in eye stalk length and cornea area as rates of increase in lengths of legs and setae, consistent with selection rather than neutral mutation as the cause of reduction of the optic structures [8]. However, the reason why selection favors smaller eyes in cave populations of *G. minus* and of *Sundathelthusa* remains unclear. In the cavefish *Astyanax mexicanus* loss of pigment results from mutations at a single gene, *oca2*, and that different populations show different mutations [8]. Further, Protas et al. 2007 [9] showed that in *A. mexicanus* the polarities of QTLs are different for eyes and pigment systems. They interpret that consistent negative polarity of QTLs for eyes is evidence of selection, but that presence of both positive and negative polarities of QTLs for pigment is evidence of neutral mutation and genetic drift. In two species of cave-dwelling planthoppers, loss of melanin pigment occurred independently and resulted from a defect at the same step in the melanin synthesis pathway, suggesting an antagonistic pleiotropic effect of molecules involved in this step on melanin production and on an unidentified adaptive trait in darkness [10]. Upregulation of the *sonic hedgehog* gene (*shh*) leads to eye degeneration in *A. mexicanus*, because increased expression of *shh* is correlated with enhanced development of taste buds [11,12]. Thus, loss of eyes in this cavefish is a pleiotropic response to selection for increased taste bud development, a probable highly advantageous character in darkness. Niven and Laughlin [13] suggested that the high energetic cost of neural tissues favors reducing sensory structures to a functional minimum to save energy, especially in environments with severe energy limitation such as caves. As stated above, however, energy conservation hypotheses are indistinguishable from hypotheses based on pleiotropy, because any selective advantage from the energy saved can only be realized by spending the energy on improving the function of other feature(s) in darkness [6].

**Figure 1:**
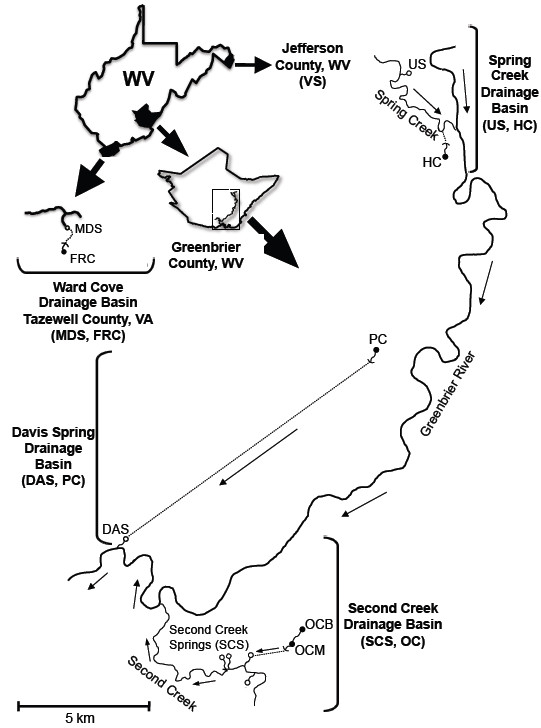
**Sampling locations for three pairs of *Gammarus minus *sister populations and one outgroup population surveyed.** Two pairs of sister populations were sampled from drainage basins in Greenbrier County, WV: the Spring Creek (US = US219 Spring, HC = Hole Cave) and Davis Spring (DAS = Davis Spring, PC = Persinger’s Cave) drainage basins. One pair of sister populations from a drainage basin in Tazewell County, VA was sampled: the Ward Cove drainage basin (MDS = Maiden Spring, FRC = Fallen Rock Cave). An outgroup surface population, Vidmont Spring (VS), was sampled from Jefferson County, WV. Cave sites are depicted as dark circles and surface sites are open circles. Dashed lines indicate subterranean conduits of unknown topology whose hydrological connection to surface resurgences have been identified through dye tracing. Arrows indicate the direction of water flow.

To further the understanding of the evolutionary mechanism behind eye loss in cave organisms, we have initiated a study of opsin visual protein evolution in the amphipod crustacean *Gammarus minus*. Aspects of the biology of this species are summarized in Culver et al. 1995 [14]. *G. minus* has an extensive geographic range, occupying a narrow band along the Appalachians plus much of the east central United States and extending west of the Mississippi River to northeastern Oklahoma. Within its range *G. minus* is a common inhabitant of carbonate springs characterized by hard, alkaline water with high conductivity [15], where it is usually the dominant macroinvertebrate species in terms of density and biomass. Adults of spring populations have large compound eyes with about 40 ommatidia, a first pair of antennae at about 45-50% of body length, brownish body pigmentation, and males reach sexual maturity at 6–9 mm body length. *G. minus* is also a common inhabitant of cave streams throughout its range. The cave streams within a subterranean drainage basin resurge onto the surface at a spring, and there is strong evidence that the cave populations within a subterranean basin originated from upstream colonization by individuals from the hydrologically connected spring population [14]. Cave populations inhabiting subsurface basins are separated from spring resurgences by phreatic sediments and porous rock when the cave stream passes below the water table [16]. The morphology of most cave populations is identical to or only slightly different from that of spring populations. Morphologically distinct populations of *G. minus* occur only in caves in two geographic areas, one in the Greenbrier Valley of southeastern West Virginia and one in the Wards Cove area of southwestern Virginia (Figure 1). Both areas have extensive cave development and large cave systems exceeding 20 km in passage length. These cave populations exhibit the classic troglomorphic syndrome [17]. They differ from spring populations in having consistently large body size with mature males attaining 10–11 mm in length, pale to bluish instead of brownish body coloration, longer first antennae that are at least 65% of body length, and greatly reduced compound eyes with only five or fewer to no discernible ommatidia.

*G. minus* is an excellent model organism to study the evolution of troglomorphy for several reasons. First, cave populations show heritable, individual variation in eye size as well as antenna size [6]. In contrast, in most other troglomorphic species all individuals lack any manifestation of eye and body pigment. Second, it is a single species with troglomorphic and non-troglomorphic populations occupying different habitats. This is important because it avoids the problem of inappropriate comparisons as most troglomorphic species are highly endemic with no close surface relatives [18]. Third, hydrological and genetic evidence [1] clearly indicate that troglomorphic populations invaded cave streams from surface spring habitats, thus showing the troglomorphies are the derived condition. Finally, evidence from allozyme variation [19] and from molecular sequence data [20] strongly indicates that multiple troglomorphic populations have evolved troglomorphy independently.

We previously conducted a survey of 15 *Gammarus minus* populations through analysis of DNA sequences from one mitochondrial gene, cytochrome *c* oxidase I (COI), and one nuclear gene, internal transcribed spacer 1 (ITS-1) [20]. Standing variation at both loci was low within populations (< 2% for COI, < 1% for ITS-1), but in accordance with the allozyme data [19] a significant degree of divergence and spatial structuring of populations was observed. Average COI sequence divergences between populations ranged from 0.4% to 10.2%, with hydrologically proximate populations exhibiting low levels of divergence, regardless of morphological differentiation. For example, the average divergence between the morphologically differentiated Second Creek Springs (SCS, surface morphology) and the hydrological proximate (3.5 km) Organ Cave (OC, cave morphology) populations was 1.43%, whereas the average divergence between SCS and the hydrologically distant (18.5 km) Davis Spring (DAS, surface morphology) populations was 4.65%, a greater than three-fold difference. A second pair of hydrologically connected populations, Maiden Spring (MDS, surface morphology) and Fallen Rock Cave (FRC, cave morphology), exhibited very low levels of genetic divergence at the ITS-1 locus (0.29%) despite significant morphological differentiation, and each of these two populations differed from all other populations by > 6% ITS-1 divergence. Therefore, different replicate pairs of surface and subterranean populations represent independent derivations of troglomorphy. As such, any concordant variation associated with troglomorphy in multiple, independently derived cave populations is much more likely to reflect a true response to selection than variation observed in only a single cave population.

Given that one of the most obvious morphological differences between surface and subterranean *G. minus* populations is in the eye (the other two being size and pigmentation), we embarked on a study of opsin visual protein evolution. In this study we characterize molecular genetic variation and patterns of gene expression in two paralogous opsin genes in four pairs of surface and subterranean *G. minus* populations to determine 1) if there is a loss of functional constraint in any of the cave lineages and 2) if there are differences opsin expression between surface and cave populations. Our study provides strong evidence for persistent purifying selection acting on the opsin genes of both the surface and cave populations, while at the same time documenting significantly reduced opsin expression in the cave populations.

## Methods

### Sampling locations, specimen collection, and preservation

Specimens were collected live from each of nine sampling locations in Tazewell County, Virginia, and Greenbrier and Jefferson counties, West Virginia, from Fall 2005 through Spring 2012. Sampling locations were selected to overlap with those from previous studies [14,19,20], and included four pairs of sister surface sites and cave sites: 1) Second Creek Springs (SCS) and Organ Cave (OC), 2) US219 Spring (US) and The Hole Cave (HC), 3) Davis Spring (DAS) and Persinger’s Cave (PC), 4) Maiden Spring (MDS) and Fallen Rock Cave (FRC), as well as Vidmont Spring (VS), a hydrologically remote surface site (Figure 1). The hydrologic connection between each pair of surface and cave sites was established by dye-tracing of the groundwater [21,22]. Specimens were either preserved immediately in 100% ethanol or transported live in coolers back to the lab, where they were maintained at 4°C in incubators containing water from the sampling location until DNA or RNA extractions were performed, usually within 2 weeks of the collection date. Both surface and cave animals that were stored prior to nucleic acid extractions were exposed to approximately 12 hours of indirect dim light per day through the glass doors of the 4°C incubators; thus, individuals from sister population pairs were treated identically.

### DNA extraction, PCR amplification, and sequencing

Approximately one fourth to one half of the posterior end of each specimen was excised for DNA extractions using clean razor blades. Qiagen DNeasy Blood and Tissue kits were used to extract genomic DNA with the following modifications of the manufacturer protocol: 30 μl of phosphate buffered saline (PBS) + 150 μl of buffer ATL were added prior to grinding the tissue for the initial step of the protocol, and a single elution was performed using 100 μl of buffer AE for the final step of the protocol.

Opsin genes were initially amplified from individuals from surface and cave populations using degenerate pairs of PCR primers designed to amplify opsin genes from a variety of crustaceans: LWF1A: 5′ -TGG TAY CAR TWY CCI CCI ATG AA -3′ [23], and Scylla: 5′-TT RTA IAC IGC RTT IGC YTT IGC RAA-3′ [24]. The forward primer LWF1A is nested within the first transmembrane domain (TM1), while the reverse primer Scylla is nested within the seventh transmembrane domain (TM7) of opsin. PCR products were cloned, and ten clones from each of two individuals from one cave (OC) and one surface population (SCS) were sequenced. Based on these comparisons we identified two divergent paralogs of the opsin gene in *G. minus* which differed by >20% at the nucleotide sequence level within individuals in both cave and surface populations. We designed *G. minus*–specific opsin primers for each of the two paralogs, which we arbitrarily designated as opsin-1 (*Ops1*, 735 bp, excluding PCR primers) and opsin-2 (*Ops2*, 747 bp, excluding PCR primers). These species–specific primers were designed from regions where there were six to eight fixed differences between the *Ops1* and *Ops2* paralogs, 10–30 base pairs downstream from the degenerate primers: Ops1-F (5′–ATC ACC ATT CTA GGC ATC C–3′), Ops1-R (5′–TC CAG ATG GTC AGC AAG G–3′), Ops2-F (5′–CTT CTC GGA TTT GCT ATC TTC G–3′), Ops2-R (5′–CC CAG ATG GTA AAG AGC G–3′).

To facilitate direct sequencing of PCR products on the LI-COR 4300 DNA Analysis System, M13F (−21) tails were added to the 5′ primer and T7 tails were added to the 3′ primer. This allowed us to use IRDye® labeled M13F and T7 sequencing primers (LI-COR Biosciences) in the subsequent cycle sequencing reactions. Species specific opsin primers were used in 25 μl PCR amplifications under standard conditions (50–100 ng genomic DNA, 100 nM each primer, 200 μM each dNTP, 10 mM Tris–HCl, 50 mM KCl, 1.5 mM MgCl_2_, 2.5 U puRE*Taq* polymerase). A total of 40 cycles of 95°C for 30 s, 47°C for 30 s, and 72°C for 30 s, were followed by a final extension at 72°C for 7 minutes. PCR products were electrophoresed on 0.8% agarose gels, excised, and purified using minElute PCR Purification kits (Qiagen).

PCR products were sequenced directly using M13F/IRD-800 and T7/IRD-700 labeled sequencing primers (LI-COR Biosciences). Both strands of DNA were sequenced with the SequiTherm EXCEL™ II DNA Sequencing Kit (Epicentre Biotechnologies), run on a LI-COR 4300 DNA Analysis System automated DNA sequencer, and read with the eSeq™ software (LI-COR Biosciences).

### Sequence analysis

Nucleotide sequences were translated into their respective amino acid sequences. Given the low levels of sequence variation within and among populations, we obtained only five unique *Ops1* and six unique *Ops2* protein sequences. We downloaded arthropod opsin protein sequences from GenBank (Additional file 1) representing a spectrum of opsin absorbance, along with several onychophoran and cephalopod opsins that were selected as outgroups. Downloaded opsins were aligned with our 11 *G. minus* opsin protein sequences using ClustalW [25]. Both alignments were uploaded to GenBank as population sets [GenBank *Ops1* Accession Numbers: JX879564 - JX879705; GenBank *Ops2* Accession Numbers: JX879485 - JX879563]. The best-fit model of protein evolution to use for subsequent maximum likelihood analyses was determined with ProtTest 3 [26]. Maximum likelihood analysis of protein sequences was conducted in PhyML 3.0 [27]. Clade support was determined through nonparametric bootstrapping (1000 replicates) in PhyML 3.0 using fixed model parameters obtained from the original data.

Nucleotide diversity (*π*) within populations and nucleotide divergence between populations were calculated in MEGA5 [28]. The PopGen modules of BioPerl were used to calculate pairwise dN/dS ratios, Tajima’s D, and to conduct coalescent simulations to test the significance of Tajima’s D [29]. McDonald-Kreitman tests were performed via the Standard and Generalized MKT website [30].

We employed a codon-based maximum likelihood approach to investigate potential variation in the dN/dS ratio over the branches of a phylogenetic tree using PAML [31]. We used a fixed input tree topology that is consistent with what is known about the hydrology [22] and genetic structure of the *G. minus* populations based on allozyme [19] as well as nuclear and mitochondrial gene nucleotide sequences [20].

### RNA extraction and quantitative real-time PCR

Quantitative real-time PCR (qRT-PCR) experiments were conducted to determine if opsin expression differed between the cave and surface populations. For an internal control gene, the 18S rRNA was amplified from *G. minus* individuals from several populations using degenerate “universal” primers. The 18S PCR products were cloned and sequenced, and a set of internal primers oriented to amplify a 440 bp fragment for qRT-PCR were designed (18SF: 5′–AGGAATTGACGGAAGGGC–3′; 18SR: 5′–GGACATCTAAGGGCATCACAG–3′). PCR products from two individuals each from one cave (OC) and one spring (SCS) population using these *G. minus*–specific 18S primers were sequenced and BLAST searched to confirm the identity of the 18S amplicon. A nested internal qRT-PCR forward primer was designed to amplify a ~300 bp fragment for the *Ops1* gene, and a different internal forward primer was designed to amplify a ~300 bp fragment for the *Ops2* gene. *Ops1* and *Ops2* qRT-PCR forward primers were designed based on the sequences obtained from the population-level analyses described above in a region where there was a fixed 10 bp difference between the two paralogs (qRT/Ops1F: 5′–T TCA TTG ATG TCA CAC AGC T–3′; qRT/Ops2F: 5′– A TCT TCT TCC TCT CAC TCC T–3′). We used the same *Ops1* and *Ops2* reverse primers for qRT-PCR as were used for the population sequencing (described above). PCR products using these opsin primers were sequenced from two individuals each from one cave (OC) and one spring (SCS) population to confirm the identity of the *Ops1* and *Ops2* amplicons.

Twenty fresh specimens from each of three pairs of hydrologically-connected populations were sampled for mRNA extractions: 1) SCS and OC, 2) HC and US, and 3) MDS and FRC. Due to the closure of PC during 2011–2012 to prevent the spread of white nose syndrome among bat populations, we were unable to obtain specimens and hence the DAS and PC population pair was not included for the gene expression analyses. Prior to mRNA extraction, individuals were photographed on both sides of the head at high resolution for subsequent measurements of eye and head surface areas. Total RNA was extracted from the heads (Ambion, RNAqueous for PCR) immediately following decapitation. To check for opsin expression outside of the head region, total RNA was extracted from the bodies of two individuals from each population. Total RNA was quantified with a microspectrophotometer (NanoVue, GE Healthcare Life Sciences), and 50 ng was used in each of two replicate oligo-dT primed cDNA synthesis reactions (AffinityScript QPCR cDNA Synthesis Kit, Agilent Technologies). Each cDNA was used to set up two replicate qRT-PCR reactions per gene using SYBR green as nonspecific fluorescent label and run on an Mx3005P QPCR System (Agilent Technologies). Ct values were calculated with the MxPro QPCR software v. 4.10, and dissociate curves for each reaction were checked to confirm that only a single PCR product was obtained. The relative expression of opsin was quantified as 2^ΔCt^, where ΔCt = (Ct_18S_ – Ct_Ops_). We corrected for differences in eye size among individuals and between populations, by dividing relative expression by the proportion of eye tissue in the mRNA preparation. Because amphipods are laterally compressed, we estimated the proportion of eye tissue as the surface area of the eye divided by the total surface area of the head.

## Results and discussion

### Characterization of *G. minus* opsin proteins

**Figure 2:**
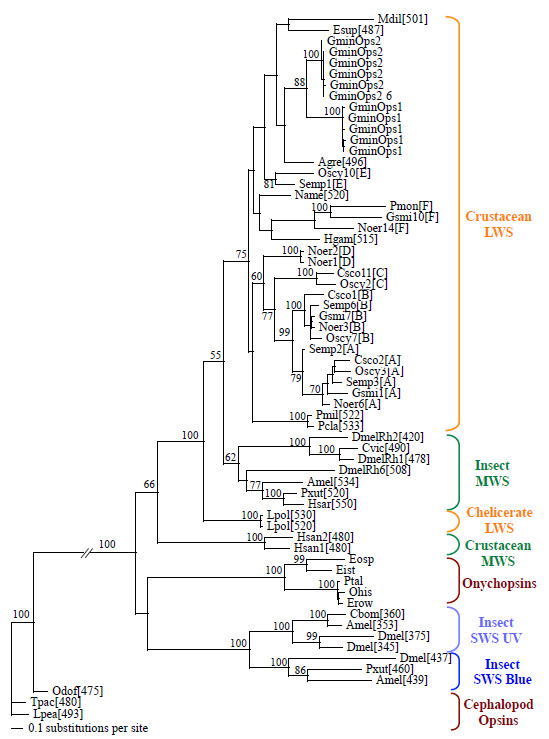
**Maximum likelihood phylogeny of *Gammarus minus *and selected arthropod and onychophoran opsin protein sequences.** Known long-wavelength-sensitive (LWS), middle-wavelength-sensitive (MWS), and short-wavelength-sensitive (SWS) opsins, along with various stomatopod and onychophoran opsin protein sequences were downloaded from GenBank (see Additional file 1) and analyzed in PhyML 3.0 using a BLOSSUM62 + Gamma best fit model of protein evolution determined with ProtTest 3. Numbers in square brackets indicate the wavelength of maximum absorbance (λmax), letters indicate the major stomatopod opsin groups identified by Porter et al. 2009 [32]. Bootstrap support (1000 replicates) are provided above nodes supported in ≥70% of the replicates. The *Gammarus minus* opsin paralogs are monophyletic, share a most recent common ancestor, and placed within the crustacean LWS group.

Sequencing of multiple clones of PCR products amplified with the degenerate LWF1A and Scylla opsin primers revealed that there are at least two divergent opsin paralogs, *Ops1* and *Ops2*, in the *G. minus* genome. On average, the *Ops1* and *Ops2* paralogs within an individual differed from one another by 19.96% at the amino acid level. In contrast, the maximum observed amino acid divergence between populations was 1.23% for *Ops1* and 1.19% for *Ops2*. Maximum likelihood analysis of the five unique *Ops1* and six unique *Ops2* amino acid haplotypes, along with various arthropod opsins revealed that both *G. minus* opsin paralogs clustered within the crustacean long wavelength sensitive (LWS) opsins (Figure 2), although bootstrap support for the monophyly of the crustacean LWS clade was moderate (75%). The monophyly of the *G. minus Ops1* and *Ops2* paralogs was supported (88%), indicating that the gene duplication event occurred after the divergence of *G. minus* from the other crustacean LWS opsins.

Our results clearly show there are two opsin paralogs in the *G. minus* genome. These opsins are homologous to other crustacean long-wavelength sensitive (LWS) opsins. Although diverged from each other, the two paralogs are more closely related to each other than to any of the other crustacean opsins. They arose through a gene duplication event following their divergence from other crustacean LWS opsins. Because there are no other amphipod opsin sequences available in GenBank (as of December 2012), we cannot rule out the possibility that the duplication event preceded the speciation of *G. minus*.

### Variation and dN/dS ratios in cave versus surface populations

Both cave and surface populations exhibited low levels (< 0.5%) of *Ops1* and *Ops2* nucleotide sequence divergence (Table 1). The average *Ops1* divergence within the cave populations (0.16%) and that within the surface populations (0.21%) was not significantly different (*P* = 0.48, Welch’s two-tailed *t*-test). Similarly, there was no significant difference between *Ops2* average divergences in the cave (0.32%) and surface (0.26%) populations (*P* = 0.61). For both genes, Tajima’s D was not significantly different from zero for any population, consistent with neutral expectations. Divergence between populations was also generally < 0.5%, with the exception of comparisons between VS and the remaining populations, where average pairwise divergences ranged up to 0.82% for *Ops1* and up to 1.67% for *Ops2* (Table 1), as expected since VS is a genetically divergent and hydrologically remote site [20]. *Ops2* is more variable than *Ops1* within six of the nine populations, and was more variable in 30 of the 36 pairwise comparisons between populations.

**Table 1:** Average % sequence divergence (uncorrected ρ) for pairwise comparisons within populations (diagonal elements in bold) and between populations

		**SCS**	**OC**	**US**	**HC**	**DAS**	**PC**	**MDS**	**FRC**	**VS**
*Ops 1*	SCS	**0.08[0.09]**	0.17	0.08	0.12	0.03	0.14	0.2	0.23	0.82
	OC		**0.20[0.09]**	0.10	0.10	0.10	0.13	0.19	0.22	0.81
	US			**0.10[0.14]**	0.01	0.01	0.07	0.09	0.12	0.72
	HC				**0.11[0.15]**	0.03	0.04	0.09	0.12	0.72
	DAS					**0.32[0.54]**	0.07	0.11	0.14	0.74
	PC						**0.15[0.25]**	0.16	0.19	0.79
	MDS							**0.35[0.61]**	0.02	0.61
	FRC								**0.16[0.19]**	0.58
	VS									**0.27[0.06]**
*Ops 2*	SCS	**0.15[0.18]**	0.02	0.11	0.22	0.03	0.30	0.25	0.20	1.38
	OC		**0.09[0.20]**	0.09	0.21	0.04	0.29	0.24	0.16	1.36
	US			**0.22[0.35]**	0.29	0.10	0.37	0.4	0.31	1.45
	HC				**0.48[0.59]**	0.20	0.49	0.51	0.43	1.57
	DAS					**0.28[0.28]**	0.30	0.32	0.24	1.37
	PC						**0.35[0.99]**	0.49	0.47	1.65
	MDS							**0.40[1.05]**	0.01	1.67
	FRC								**0.34[0.64]**	1.51
	VS									**0.18[0.00]**

*We used modified dN/dS, where dN/dS = dN/dS((dS + 1)/S). This measure remains defined when dS = 0.

Numbers in sequence brackets indicate the average pairwise dN/dS ratios* within populations.

The ratio of nonsynonymous to synonymous substitutions, dN/dS, was calculated using the method of Stoletski and Eyre-Walker 2011 [33], which allows dN/dS to be calculated when dS = 0, for all pairwise comparisons within populations. For both *Ops1* and *Ops2*, the average dN/dS ratio was < 1 for nearly all populations, the only exceptions being PC and MDS populations for *Ops2*, where the dN/dS ratio was very close to 1 (Table 1). No significant differences in the average pairwise dN/dS ratio of cave and surface populations were detected for *Ops1* (cave = 0.17, surface = 0.35; *P* = 0.28, Welch’s two-tailed *t*-test), nor for *Ops2* (cave = 0.61, surface = 0.47; *P* = 0.60). We also performed McDonald-Kreitman tests (MKTs) of selective neutrality for the four population pairs, and well as MKTs for each population versus VS. All MKTs for both *Ops1* and *Ops2* were not statistically significant. Taken together, these results indicate that neither opsin gene is under relaxed functional constraint in the cave populations.

**Figure 3:**
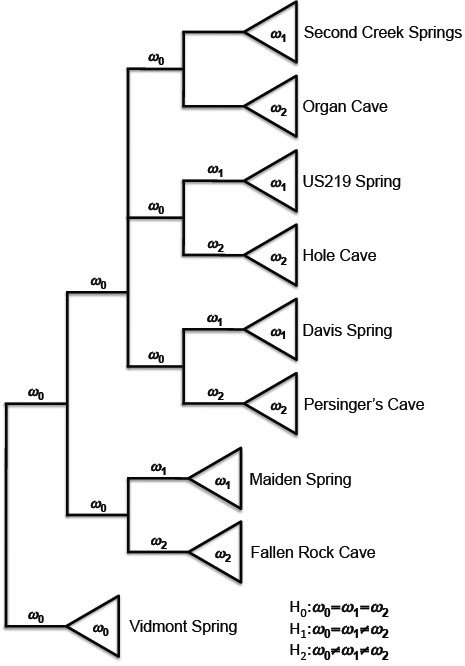
**Tree topology and dN/dS ratio (*ω*) branch parameters used in ML tests of selective constraint.** A maximum likelihood analysis of codon-based models (PAML) was employed to test for possible differences in selective constraint among cave and surface populations. The dN/dS ratio in ancestral lineages is *ω*_0_, the dN/dS ratio in surface lineages is *ω*_1_, and the dN/dS ratio in cave lineages is *ω*_2_. Under the one-ratio model there are no differences in selective constraint and *ω*_0_ = *ω*_1_ = *ω*_2_. Under the two-ratio model the ancestral and surface lineages have the same dN/dS ratio whereas the cave lineages have a unique dN/dS ratio such that *ω*_0_ = *ω*_1_ ≠ *ω*_2_. The three-ratio model allows for three unique dN/dS ratios in the ancestral, surface, and cave lineages such that *ω*_0_ ≠ *ω*_1_ ≠ *ω*_2_. See Table 2 for parameter estimates and likelihood ratio test results.

It is often difficult to detect the relaxation of functional constraint using the pairwise approach to estimating the dN/dS ratio since the approach averages selective pressure over the entire evolutionary history of the lineages in question [34]. The power to detect differences in selective pressure is increased using a maximum likelihood approach, which allows the dN/dS ratio to vary over the branches of a phylogenetic tree. The estimation of selective pressure can be combined with a formal statistical approach to hypothesis testing, the likelihood ratio test (LRT), to determine if there is any variation in the opsin dN/dS ratios associated with habitat. The codon-based model in PAML computes the maximum likelihood estimate of nonsynonymous (dN) and synonymous (dS) substitution rates (dN/dS = ω) on a given tree, as well as the overall likelihood (*L*) score for the tree under a given model [31]. We examined three models: 1) a “one-ratio” model in which all branches on the tree have the same substitution rate ratio (ω_0_), 2) a “two-ratio” model in which the derived cave lineages have a unique substitution rate ratio (ω_2_) which is distinct from the background and derived surface ratios (ω_0_ = ω_1_, respectively), and 3) a “three-ratio” model in which each of the background, derived surface, and derived cave lineages have distinct ratios (ω_0_ ≠ ω_1_ ≠ ω_2_) (Figure 3).

If the opsin genes in the cave lineages were under relaxed functional constraint, under the three-ratio model we would expect ω_2_ to be greater than ω_0_ and/or ω_1_. Furthermore, we would expect that the *L* scores under the two-ratio and/or three-ratio models would be significantly greater than the *L* score under the one-ratio model. Although the three-ratio provided a better fit to the data than either the one- or two-ratio models for the *Ops1* gene, there was no evidence of a relaxation of functional constraint in the cave lineages since ω_2_ < ω_1_ in the two- and three-ratio models (Table 2). In contrast, the results from PAML analyses of the *Ops2* data were consistent with some relaxation of functional constraint in the cave lineages as ω_2_ > ω_1_ in both the two- and three-ratio models. However, in no case was ω_2_ found to be > 1, suggesting that the evolution of the *Ops2* in cave lineages is still governed primarily by purifying selection, although perhaps to a lesser degree than in the surface lineages.

**Table 2:** **Parameter estimate, likehoods, and likehood ration test (LRT) results for three models of coding sequence evolution of the *Ops1 *and *Ops2 *genes**

**Gene**	**Model**	**ω**_**0**_	**ω**_**1**_	**ω**_**2**_	***L***	2*Δ*L*	***P***
*Ops1*	One-Ratio, H_0:_ ω_0=_ω_1=_ω_2_	0.1681	0.1681	0.1681	−1851.5071		
	Two-Ratio, H_1:_ ω_0=_ω_1≠_ω_2_	0.1807	0.1807	0.1506	−1851.4025	0.2093	0.6473
	Three-Ratio, H_2:_ ω_0≠_ω_1≠_ω_2_	0.0195	0.2756	0.1507	−1845.6865	11.4325	0.0007
*Ops2*	One-Ratio, H_0:_ ω_0=_ω_1=_ω_2_	0.3034	0.3034	0.3034	−1958.1442		
	Two-Ratio, H_1:_ ω_0=_ω_1≠_ω_2_	0.1589	0.1589	0.5334	−1952.6264	11.0356	0.0009
	Three-Ratio, H_2:_ ω_0≠_ω_1≠_ω_2_	0.0426	0.2208	0.5335	−1950.0496	5.1536	0.0232

See Figure 3 for the topology and branch-specific non-synonymous to synonymous rate ratios (ω). The one-ratio model assumes a single ω on all branches, the two-ratio model allows for a background rate and a cave-specific rate, and the three-ratio model allows for a background rate, a derived surface rate, and a derived cave-specific rate.

There is no loss of functional constraint in the opsin genes of cave populations of *G. minus*, and this appears to be a common finding in cave-adapted organisms. Crandall and Hillis 1997 [35] found no difference in the evolutionary rates of the rhodopsin gene between surface crayfish and eyeless crayfish species that differed in presumed durations of isolation in the subterranean habitat, and suggested that rhodopsin may be functional in these blind species. In the cave–adapted beetle *Ptomaphagus hirtus* highly reduced but persistent visual capacities were discovered by recovering transcripts of the phototransduction protein machinery in the adult head transcriptome [36], suggesting that the light detection machinery may play a role in regulation of circadian or other oscillatory processes. This beetle is similar to cave populations of *G. minus* in that it retains a residual lens structure in the degenerated eye and it is photonegative and thus able to detect light. Cave populations of *G. minus* (OC and PC) are also able to detect light but exhibit greatly reduced negative phototaxis compared to surface populations (SCS and DAS) [37]. It is, however, possible that opsins in *G. minus* may play a direct or indirect role in regulating oscillatory processes as well, because the cave streams inhabited by *G. minus* experience seasonal influx of allochthonous organic detritus, which is assumed to be the ultimate source of energy in an aphotic environment in the absence of chemosynthesis [1]. Teleost multiple tissue (TMT) opsin and melanopsin have been demonstrated to function as extra-retinal photoreceptors, and they may play a role in a peripheral clock in the blind Somalian Blind Cavefish *Phreatichthys andruzzii*[38]. Similar to *P. hirtus* and *G. minus*, *P. andruzzii* also exhibits a phototactic response [39].

In *Drosophila* larvae, an opsin protein has recently been shown to function in thermodiscrimination [40]. Interestingly, larvae containing a deletion of the opsin *ninaE* gene exhibited an impaired ability to discriminate between 18°C, their preferred temperature, and 24°C, whereas the thermodiscrimination phenotype was rescued upon addition of the wild-type *ninaE* transgene. In another recent study in *Drosophila*, four opsin paralogs were found to be expressed in the mechanosensory cells of the antennal ear and were required for hearing [41]. Thus, we cannot rule out the possibility that a light-independent function of opsin is responsible for its conservation in cave populations.

It has also been suggested that genes or entire genetic pathways underlying the loss of structures may be maintained because they serve multiple functions or pathways, and can be re-expressed subsequently [42]. Examples of the resurrection of “lost” structures include the evolution of the compound eye in myodocopid ostracod crustaceans [43], the induction of enamel organs in chick oral epidermis [44], and the development of wings in stick insects [45]. Although a common theme, the lack of evidence for loss of functional constraint in the opsin genes of cave-adapted organisms is not universal. For example, a recent study of amblyopsid cavefishes documented at least three instances of loss of functional constraint due to indels and premature termination codons [46]. However, the cavefish in this study are members of different species that have been diverging over the past 10 million years, a much longer time frame than the divergence of intraspecific sister population pairs in *G. minus*. It is possible that cave species may lose color-vision related opsins, while the other opsin paralog(s) remains conserved. In this case, one would predict that the LWS opsins would most likely remain conserved because they are maximally sensitive to low light levels.

### Gene expression

Given the lack of evidence for loss of functional constraint in the cave populations, any observed differences between cave and surface populations in the level of opsin expression would be particularly interesting. For example, down-regulation of opsin expression in cave populations would be consistent with the notion that adaptive morphological evolution is due primarily to changes in regulatory regions, rather than changes in the coding sequences of structural genes, so as to minimize pleiotropic effects [47,48].

**Figure 4:**
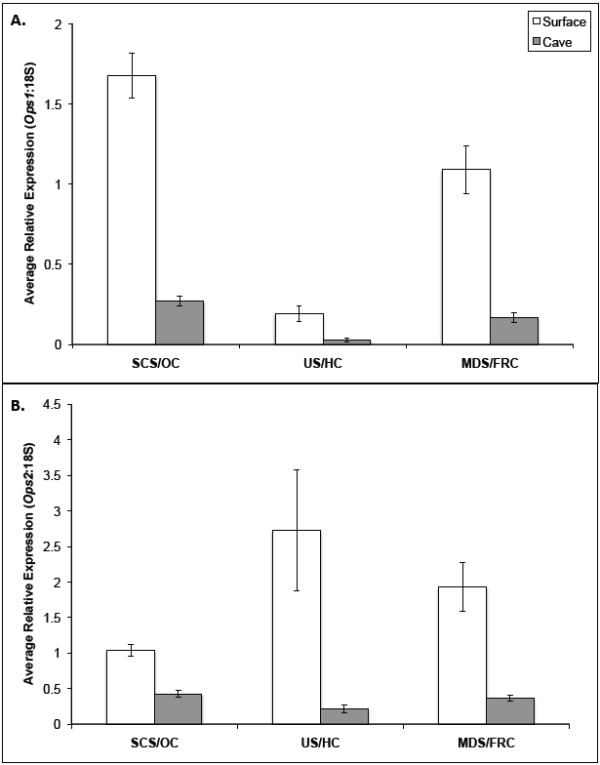
**Comparisons of opsin expression in sister pairs of surface and cave populations. A**. Average relative expression of *Ops1* to 18S as measured by qRT-PCR (*n* = 20 individuals per population). **B**. Average relative expression of *Ops2* to 18S as measured by qRT-PCR (*n* = 20 individuals per population). Error bars depict standard error of the mean. All comparisons between sister populations were statistically significant (*P* < 0.01, nested ANOVA, Table 3), indicating a reduction in opsin expression in the cave populations.

For both the *Ops1* and *Ops2* genes, the average relative expression of opsin genes (Ops:18S) were significantly higher in each of the surface members of the population pairs (5.6 × 10^-11^ < *P* < 0.0085, nested ANOVA, Table 3). In each pair of populations there was at least a six-fold difference in average relative *Ops1* expression between the surface and cave populations (Figure 4A: SCS = 1.68, OC = 0.27; US = 0.19, HC = 0.03; MDS = 1.38, FRC = 0.17). Differences in the relative expression of *Ops2* among surface and cave populations were less pronounced than for *Ops1*, yet expression in surface populations was still significantly higher in each pair (Figure 4B: SCS = 1.04, OC = 0.43; US = 2.73, HC = 0.21; MDS = 1.93, FRC = 0.37). For both genes there was also substantial variation in expression from individual to individual within populations (P < 0.01, nested ANOVA, Table 3). For five of the six populations assayed, *Ops2* relative expression was significantly greater than *Ops1* expression. In MDS, the one population where this difference was not significant, *Ops2* expression was also greater than *Ops1* expression. This difference is somewhat surprising given that we observed less sequence variation in *Ops1* genes, which appear to be evolving under stronger purifying selection than *Ops2*. Since the same cDNA was used in qRT-PCR of all three genes (*Ops1*, *Ops2*, 18S) for each specimen, and since the qRT-PCR was performed simultaneously for all three genes in the same experiment for each specimen, the consistent differences between *Ops1*/*Ops2* relative expressions are not likely due to experimental error. Given the contradictory pattern that the more conserved paralog was more weakly expressed, it is hard to draw any conclusions about possible differential functions of the two paralogs. There were no detectable levels of expression using cDNA prepared from body tissue mRNA for either opsin gene in any of the six populations (*i.e.*, Ct values were indistinguishable from the negative controls), indicating that the expression of both opsin paralogs was limited to the head region.

**Table 3:** Comparison of gene expression differences among sister populations and among individuals within populations

**Gene**	**Comparison**	**Source of variation**	**df**	**SS**	**MS**	**F**	***P***	
*Ops1*	SRC vs OC, uncorrected	Among Populations	1	148.894	148.894	188.553	5.600E-11	***
		Among Individuals	38	30.007	0.790	5.184	0.000320	***
		Trial	40	6.093	0.152			
	SRC vs OC, corrected for eye size	Among Populations	1	2277.056	2277.056	6.166	0.023105	*
		Among Individuals	38	14033.710	369.308	4.123	0.001484	**
		Trial	40	3583.039	89.576			
	US vs. HC, uncorrected	Among Populations	1	0.533	0.533	9.891	0.005599	**
		Among Individuals	38	2.046	0.054	3.736	0.002749	**
		Trial	40	2393.263	59.832			
	MDS vs. FRC, uncorrected	Among Populations	1	29.412	29.412	13.854	0.001560	**
		Among Induviduals	38	80.673	2.123	16.813	2.307E-08	***
		Trial	40	5.051	0.126			
	MDS vs. FRC, corrected for eye size	Among Populations	1	2182.124	2182.124	2.994	0.100701	ns
		Among Individuals	38	27699.101	728.924	14.557	8.271E-08	***
*Ops2*	SRC vs. OC, uncorrected	Among Populations	1	39.946	39.946	47.245	0.000002	***
		Among Individuals	38	32.129	0.846	6.270	0.000081	***
		Trial	40	5.394	0.135			
	SRC vs. OC, corrected for eye size	Among Populations	1	8653.153	8653.153	15.578	0.000945	***
		Among Individuals	38	551.633	14.517	24.123	8.650E-10	***
	US vs. HC, uncorrected	Among Populations	1	126.647	126.647	8.724	0.008499	**
	US vs. HC, corrected for eye size	Among Populations	1	74198.874	74198.87	6.09432	0.023806	*
		Among Individuals	38	462653.1	12175.08	17.4017	1.69559E-08	***
		Trial	40	27986.008	699.6502			
	MDS vs. FRC, uncorrected	Among Populations	1	48.912	48.912	21.098	0.000226	***
		Among Individuals	38	88.099	2.318	17.337	1.753E-08	***
		Trial	40	5.349	0.134			
	MDS vs. FRC, corrected for eye size	Among Populations	1	64.220	64.220	0.053	0.821281	ns
		Among Individuals	38	46445.260	1222.244	7.781	0.000016	***
		Trial	40	6283.156	157.079			

The statistical significance of differences in expression values were tested for using a nested ANOVA. Significance: *P < 0.05, **P < 0.01, ***P < 0.001.

When scaled for differences in eye surface area between the surface and cave populations, the difference in expression remained significant for the *Ops1* gene in the SCS/OC comparison (*P* < 0.05), and for the *Ops2* gene it was significant for both the SCS/OC (*P* < 0.001) and US/HC (*P* < 0.05) comparisons (Table 3). This suggests that the reduced expression in these cave populations is not simply the result of a smaller eye size (= fewer ommatidia) and thus fewer opsin expressing cells, but that these opsin genes may be differentially regulated in the surface and cave populations. However, the estimation of expression levels normalized by eye size is only approximate and may not be truly reflective of cell specific opsin expression. Compound eyes of cave populations appear disorganized compared that of surface populations. No organized rhabdomere, consisting of a lens and a rhabdom surrounded by a reflecting pigment shield, is observed in any of the cave populations. Different cave populations have lost a different set of these components, with the Organ Cave population having lost all of these components [14]. The reduced opsin expression in cave populations, combined with a lack of evidence for loss of functional constraint, supports the view that adaptive evolution tends to proceed via changes in *cis* regulatory regions rather than via changes in the coding sequence of structural genes [47,48].

The concordantly lowered expression level of both opsin genes in cave populations of *G. minus* compared to sister surface populations is consistent with an as of yet unspecified pleiotropic function of opsin proteins, which may require a threshold level of expression. Aspiras et al. (2012) also found a consistently lowered expression of the eye development gene *hedgehog* in the same cave populations of *G. minus* compared to the same sister surface populations, but no difference in expression of the upstream genes *pax6*, *dachshund*, and *sine oculis*[49]. Their results echo the finding that upregulation of the *shh* gene leads to decreased eyes but increased taste bud development in the cave fish *Asytanax mexicanus*[11,12]. They pointed out that although there was downregulation in an invertebrate system compared to upregulation in a vertebrate system, both involved the same homolog of *shh*.

## Conclusions

The molecular signature of both opsin paralogs did not reveal any obvious relaxation of selective constraint, such as premature termination codons, indels, or elevated dN/dS ratios in the cave populations. Any or all of the three following explanations may account for our observations: 1) the sister populations have not been diverging long enough for such substitutions to accrue in the cave populations, 2) these opsin genes perform an alternate function unrelated to vision, and/or 3) these opsins retain some visual function in peripheral photoreceptors. Given the signature of persistent purifying selection, combined with the reduced but measureable expression of both paralogs in multiple independently derived cave populations, our results suggest that phototransduction proteins such as opsins may retain their function in cave-adapted organisms because they play an important role in peripheral photoreceptors or in other as of yet unidentified cellular processes and functions. In principle, these non-visual functions could be identified through experiments using RNAi to eliminate opsin expression in surface populations to determine what affect this would have on non-visual functions such as oscillatory behavior or temperature preference.

Our finding that there were differences in expression levels, but not in the coding sequences, of cave versus surface populations makes sense when considering the pleiotropic effects of mutations in coding versus regulatory regions. A mutation in a coding region of a gene will affect the function of the encoded protein in all tissues, whenever it is expressed. In contrast, a mutation in a single *cis*-regulatory element will only affect the expression of that gene in the domain under the control of that regulatory element. In this way, the diversification of individual regulatory elements allows expression to be modulated in a tissue– and/or temporal–specific manner without altering gene function. Given the lack of evidence for loss of functional constraint in both *G. minus* opsin genes, it appears possible that *cis* regulatory changes underlie the morphological differences between the cave and surface populations.

Our results are similar to the finding that the enzyme catalyzing reactions downstream of the defect in the melanin synthesis pathway in cave adapted insects remain functional because they also play an important role in the innate immune response and in wound healing in arthropods [50-52]. The reduced expression of opsin paralogs in cave populations, perhaps driven by increased *hh* expression, may be a pleiotropic consequence of selection for other cave-adapted structures similar to the pleiotropic effects of *shh* expression on eye size and taste bud development in cave fish [11,12]. Interestingly, structures analogous to a lateral line system have been described from another species of *Gammarus* amphipod [53]. Comparison of such structures in surface and cave populations of *G. minus* may point to a potential pleiotropic function of lowered opsin expression.

## Competing interests

The authors declare that they have no competing interests.

## Authors’ contributions

DBC participated in the conception and design of the study, helped carry out sequencing of the opsin genes, performed RNA extractions and qRT-PCR for the expression studies, conducted the phylogenetic and statistical analyses, and drafted the manuscript. SS performed genomic DNA extractions and helped carry out sequencing of the opsin genes. DWF participated in the conception and design of the study, collected all specimens from the field sites, and helped to draft the manuscript. All authors read and approved the manuscript.

## Supplementary Material

Additional file 1 GenBank accession numbers of arthropod, cephalopod, and onchophoran opsin sequences used in maximum likelihood analysis of opsin protein sequences. Click here for file
